# ecolo-zip: A global, rich and granular characterization of biogeophysical ecology for 1.5 million postal codes

**DOI:** 10.1038/s41597-023-02579-0

**Published:** 2023-09-29

**Authors:** David Willinger, Friedrich M. Götz, Stefan Stieger

**Affiliations:** 1 Department of Psychology and Psychodynamics, Karl Landsteiner University of Health Sciences, Krems an der Donau, Austria; 2 Department of Psychology, University of British Columbia, Vancouver, BC Canada

**Keywords:** Environmental social sciences, Sociology, Ecology

## Abstract

The inescapable fact that human life is perpetually embedded in a tangible biogeophysical environment – and the consequences that this has for individuals and societies – have long fascinated scholars of all backgrounds. Technological progress and the advent of big data have spurred ever-more precise attempts to quantify our biogeophysical environments. However, many such datasets lack spatial granularity, global coverage, content depth, or accessibility. Here, we introduce *ecolo-zip*, a novel geospatial dataset that provides a granular-yet-global, parsimonious-yet-rich ecological characterization of over 1.5 million postal codes across 94 countries and regions. Combining two large-scale satellite image resources (ASTER; SRTM, ICC = 0.999) and a customized geospatial sampling model, we provide high-resolution indicators of physical topography (elevation, mountainousness, distance to sea), vegetation (normalized difference vegetation index), and climate (surface temperature). With this resource – featuring methodological details, visualizations, and application suggestions – we hope to contribute towards understanding the multi-faceted interactions between humans and their environments.

## Background & Summary

Human life does not exist in a vacuum^1^. Wherever we go, we are embedded in a biogeophysical ecology that we inevitably interact with and that provides the backdrop against which all of our personal, social, and cultural behaviors occur^2^. It is this ubiquity and centrality in our lives that makes ecology such an integrative, transcending force at the nexus of social and physical sciences^3,4^. It is thus not surprising that researchers have long been studying the multi-faceted relationships between environmental factors and human behaviors from a diverse array of disciplinary perspectives^5^. This includes proximate fields such behavioral ecology^6^, environmental psychology^7^, ecological psychology^8^, and socioecology^9^ but also spans archaeology^10^, anthropology^10^, economics^11^, epidemiology^12^, geography^13^, history^10^, politics^14,15^, sociology^16^, tourism^17^, and urban planning^18^. A common challenge and shared *sine qua non* that unites all of these diverse fields of study is the question of how to best capture, measure, and quantify the surrounding biogeophysical ecology in which all human behaviors unfold. That is, across disciplines there is a hunger for a rich and granular characterization of our biogeophysical ecology. In practice, however, existing datasets are often (a) not very granular, (b) not very global, (c) not very detailed, (d) not very easy to obtain, (e) not very easy to use, or any combination of above.

In an effort to push the envelope and offer researchers interested in the assessment of biogeophysical ecologies data at a granular-yet-global scale, here we release *ecolo-zip*, a newly-created geospatial dataset that provides rich, and detailed information on various indicators of three key elements of the biogeophysical ecology (i.e., physical topography, vegetation, climate) for over 1.5 million postal codes across 94 countries and regions. Powered by two large-scale satellite image data resources (Advanced Spaceborne Thermal Emission and Reflection Radiometer, ASTER; Shuttle Radar Topography Mission, SRTM), our novel geospatial dataset provides cross-validated, highly accurate ecological descriptors at the most fine-grained spatial level that is consistently used across the globe: postal codes. In creating the dataset, we made an effort to compile diverse indicators of physical topography (i.e., elevation, mountainousness, distance to sea), vegetation (i.e., normalized difference vegetation index), and climate (i.e., land surface temperature). This choice was motivated by the aim to select relevant ecological features that – collectively – (a) offer a comprehensive and rich, yet parsimonious description of humans’ biogeophysical ecology and (b) have previously garnered interest across scientific literatures and are thus likely to boost ongoing – and inspire future – work and be of direct, practical value to scholars of different backgrounds.

To illustrate, psychologists are studying physical topography as an ecological determinant of personal psychological characteristics, such as personality^19–21^ and values^22^. Political scientists are highlighting the role of mountainous landscapes in the outbreak and duration of civil wars^14,15,23^. Archaeologists, anthropologists and historians are investigating highland geography to understand when, how, and why historical trade routes, such as Asia’s silk roads emerged^10^. Tourism scholars are documenting mountain-specific resource characteristics to develop targeted tourism concepts^17,24^. Urban planners stress the influence of topography on the development, maintenance, and effectiveness of public transport networks^18^, and geographers consider physical topography to better understand where people are likely to move and settle^25^.

Likewise, vegetation has been enjoying steady attention from researchers of many different disciplines. Epidemiologists and medical researchers study vegetation and its implications for individual and population health behaviors (e.g., exercise) and outcomes (e.g., obesity)^12^. Psychologists stress the benefits of greener environments for personal happiness^26^. Sociologists elucidate the associations between ambient vegetation and crime rates in urban areas^16^ and urban planners make it a key consideration for urban development and renewal projects^27,28^.

A similar picture emerges for the study of temperature. Economists and agronomists study the effects of temperature for local economies and agricultural yields^11^. Psychologists link temperature clemency to personality traits^29–31^ and political scientists study the effect of temperature changes to voter turnout in presidential elections^32^.

Taken together, we believe that our new data resource – which provides detailed records of physical topography, vegetation, and temperature for 1.5 million postal codes from 94 countries and regions across all continents – has the potential to improve and perhaps even transform our understanding of the multi-faceted ways in which biogeophysical ecologies shape individuals and the societies they live in. In the following, we (a) introduce the underlying source datasets, (b) explain the methodological steps that were taken to extract the new data, (c) describe the resultant ecological indicators, outline their technical validation, and (d) close with usage notes.

## Methods

### Source data

*Ecolo-zip* is built upon two primary sources of ecological raw data (i.e., elevation data from the Shuttle Radar Topography Mission, SRTM; elevation and emissivity data of the Advanced Spaceborne Thermal Emission and Reflection Radiometer instrument, ASTER) in combination with a dataset that comprises global geographical identifiers (postal codes and geo-coordinates) from *geonames.com*.

#### SRTM

The digital elevation model (DEM) of the SRTM has been the gold standard in global terrain mapping for nearly a decade after its release in 2000^33^. It is based on the data of an 11-day shuttle mission that was launched in February 2000 and provided freely available raster images for terrain analysis in a resolution of 3 arc seconds (≈ 90 m). The elevation data for the SRTM DEM was calculated using the radar interferometry method^34^ and provides a coverage of about 80% of the earth’s land surface falling on latitudes between 60°N and 56°S (WGS84, also known as WGS 1984, refers to the World Geodetic System, which encompasses the definition of the coordinate system’s fundamental and derived constants, a description of the associated World Magnetic Model, the normal gravity Earth Gravitational Model, and a current list of local datum transformations; it is the gold standard for use in geodesy, cartography, and satellite navigation including GPS). Despite its widespread use and unprecedented accuracy at the time of release, voids due to inaccurate radar reflections in mountainous areas because of slope, aspect, or height^35–37^, and limited data coverage for some Northern European and North American states and territories have been crucial limitations of the dataset.

#### ASTER

At a similar time – in December 1999 –, the National Aeronautics and Space Administration’s (NASA) Terra satellite was launched, carrying the ASTER instrument which collected data for eight years with global coverage of 99% of the earth’s landmass encompassing latitudes between 83°N and 83°S. For a detailed account of how the ASTER data were collected, please refer to the work of Hulley *et al*.^38^. We used the source dataset provided by NASA as 1°-by-1° tiles that included all scenes that were cloud-masked, stacked, averaged, and scrubbed (removing residual bad values, outliers, or anomalies) and distributed with a resampled spatial resolution of 100 m (10.5067/Community/ASTER_GED/AG100.003).

In contrast to the SRTM mission, the different subsystems of the ASTER instrument allow researchers to assess not only elevation, but also important environmental indices derived from spectral contrasts. This additional data is distributed via the ASTER Global Emissivity Dataset which was released in 2014^38^. Here, *emissivity* is the intrinsic property of a surface defined by its material composition which is invariant to meteorological conditions^38^. The source product includes not only the mean and standard deviation of emissivity, but, more importantly, emissivity-derived measures such as the normalized difference vegetation index (NDVI), the land surface kinetic temperature, and a so-called land-water mask (i.e., an estimate of the percentage of water across a surface area). Again, millions of (cloud-free) images (i.e., scenes) from a period of nine years (2000–2008) were used to localize and aggregate the emissivity resulting in a global map. The land surface temperature and emissivity layers of the source dataset have been computed using the ASTER Temperature Emissivity Separation algorithm^39^.

**Fig. 1:**
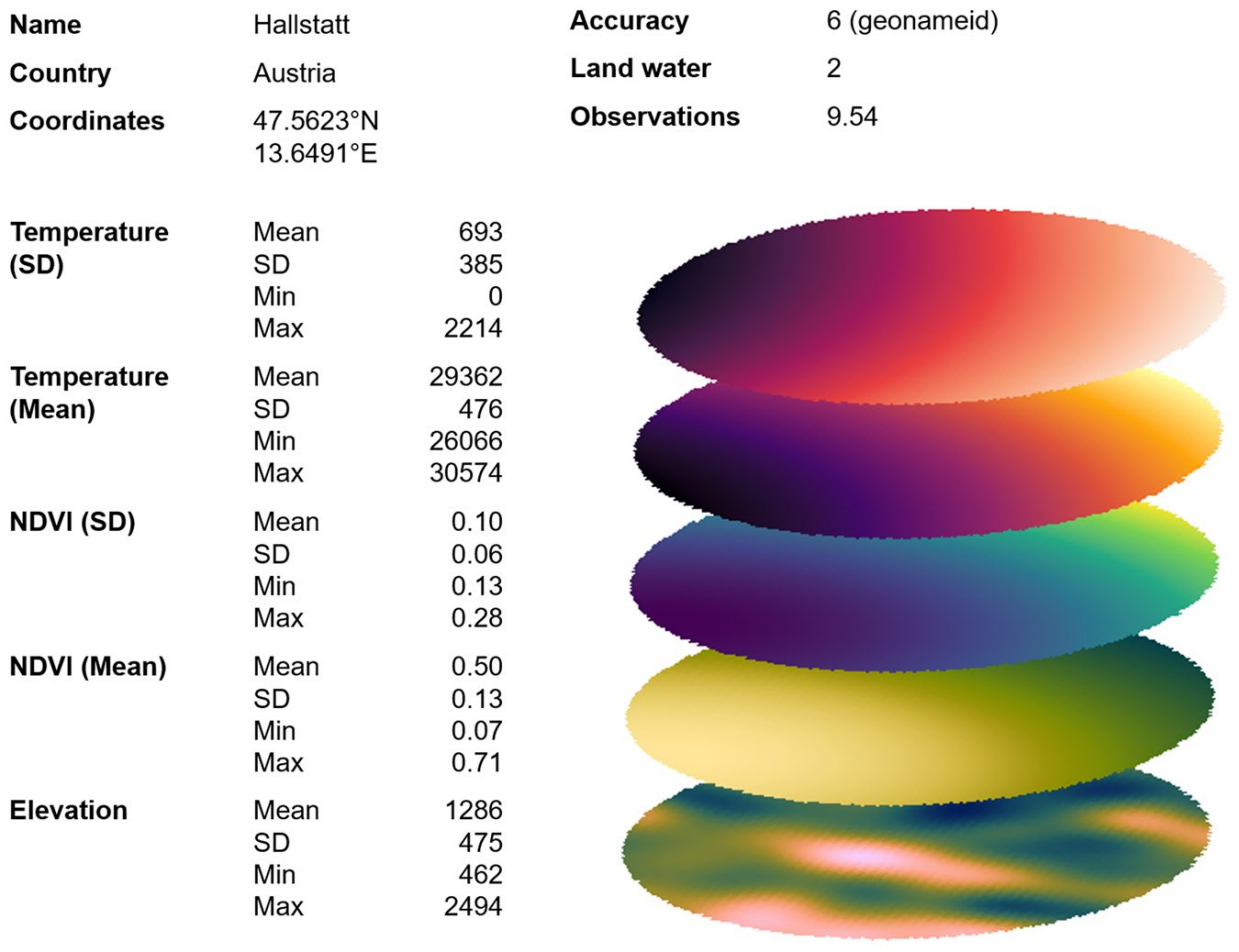
Example of different layers for the municipality of Hallstatt, Austria. For each entry in the *geonames* database, we aggregated the values for the 140 sampling points regarding the mean, standard deviation (SD), minimum, and maximum, as well as counts of water occurrences and average observations.

The layers that represent the base for the dataset provided in this work are provided in Supplementary Table 1 and Fig. 1.

#### Postal codes

The source dataset of postal codes includes estimates of coordinates of longitude and latitude in WGS84, administrative names and ISO country codes, and a coordinate accuracy classification. It is maintained by the gazetteer *geonames.com*, a globally available database and “dictionary” for toponyms. *Geonames.com* provides geo-coordinates for postal codes based on spatial administrative information (e.g., official government data) that show good accuracy^40–42^ and their utility has been demonstrated across various prior studies^43–46^.

### Geospatial sampling model based on postal code geo-coordinates

For our current purposes, the data aggregation to the postal code level was implemented through a series of four computational steps based on our custom geospatial model (see below). That is, for each postal code, we (a) retrieved the centroid’s geo-coordinates; (b) defined sampling points captured within a 20-mile radius around the postal code’s centroid; (c) retrieved topographic and environmental data for each sampling point; and (d) calculated means, standard deviations, minima, and maxima for each variable across all sampling points. To better illustrate the resulting data layers that were aggregated to create the final *ecolo-zip* data, Fig. 1 visualizes the process for the example of the Austrian City of Hallstatt. Supplementary Table 1 summarizes the spatially aggregated biogeophysical variables available in the *ecolo-zip* dataset.

### Definition of the geospatial model

**Fig. 2:**
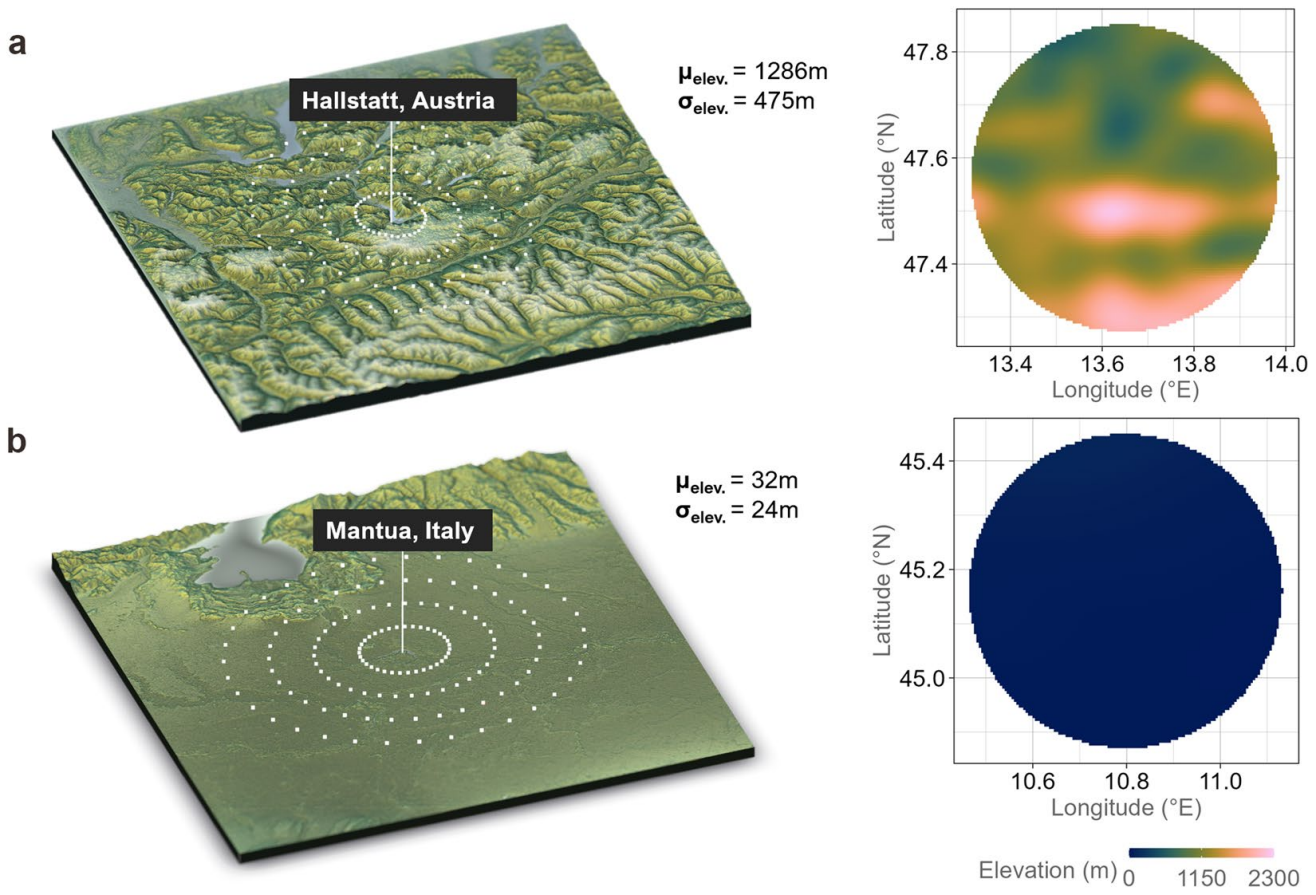
Elevation models with 140 sampling points. (**a**) Hallstatt, Austria, in the Alps and (**b**) Mantua, Italy, in the Padan Plain. White points reflect the estimated latitude and longitude of sampling points for the geospatial sampling model for calculating the estimated metrics (mean, standard deviation, minimum, maximum).

To capture the lived-in ecology of the residents of any specific postal code, we defined a geospatial model spanning a circle with a 20-mile radius around the centroid of each respective postal code. This distance was chosen as a reasonable perimeter of the model, as it reflects common commuting distances in the USA and Europe^47,48^ and has been shown to aptly capture people’s primary day-to-day living environments^19,22^. For each postal code we drew circles in 5-mile increments starting from the centroid’s longitude *λ*_0_ and latitude *ϕ*_0_ until the outer circle was reached (see also Fig. 2). That is, we drew circles with radii, of *r* = 5, 10, 15 and 20 miles. Sampling points were defined every 10°, that is, *θ* = {0°, 10°, 20°, …, 350°}, along each circle resulting in 35 (points per circle) × 4 (circles) = 140 available points for each postal code. The formula for retrieving the geo-coordinates of each point was given by

1 $$\lambda ={\lambda }_{0}+r\ast \cos (\theta )$$

2 $$\phi ={\phi }_{0}+r\ast \sin (\theta )$$

where *λ*_0_ represents the centroid’s longitude and *ϕ*_0_ represents the centroid’s latitude.

The longitude and latitude for each sampling point were calculated and used for cross-referencing in the ASTER and SRTM datasets to subsequently calculate our variables of interest for each postal code.

The final dataset – featuring 1.5 million postal codes across 94 countries and regions – consisted of ≈220 million sampling points. As it is practically – if not theoretically – infeasible to manually look up satellite data in online databases for all ≈220 million individual sampling points, we created a local repository of satellite data provided by the NASA data pool retrieved from the ASTER module on the Terra satellite. We downloaded the 1°-by-1° tiles of the ASTER source dataset (retrieved on November 4, 2021, from https://e4ftl01.cr.usgs.gov/ASTT/AG100.003/2000.01.01/) containing all source values needed for all postal codes (10,292 tiles, 422 GB) on a local machine. That way, we could process the data locally utilizing an in-house lookup routine written in *C*++ resulting in a major performance advantage. For each postal code, we aggregated the data for the variables of interest (elevation, normalized difference vegetation index, land surface temperature, land-water mask, observations) and calculated means, standard deviations, minima and maxima, whenever applicable (Supplementary Table 1).

A similar approach was taken for the SRTM elevation data (NASA and CGIAR Consortium for Spatial Information, retrieved on November 4, 2021, from https://srtm.csi.cgiar.org/), which are distributed via *datasciencetoolkit.org*. The datascience toolkit webserver was installed on a local machine to retrieve the elevation data for each sampling point to maximize lookup performance. The SRTM elevation data were collected locally for all sampling coordinates of the respective postal codes and aggregated by mean, minimum, maximum, and standard deviation.

### Data processing software

All calculations were performed on computers running Windows 10 (x64). ASTER datasets were processed using *R*^49^ utilizing the packages *raster* and – to increase performance of data retrieval – *Rcpp*^50^ providing an interface for low-level programming in *C++*. Processing involved reading in the list of sample points (latitude, longitude) for each postal code, retrieving the corresponding variable values from the data tile and storing the information. Subsequently, we used SPSS (v27, IBM) to aggregate the data by postal code using the software’s built-in functions for the computation of means, standard deviations, minima, and maxima. To generate the visualizations we utilized the *R* packages *rayshader*^51^, *plot3D* and *layer* (https://github.com/marcosci/layer).

## Data Records

All data can be freely accessed in the repository^52^ (10.17605/osf.io/wcjad). There are two files for the dataset AllCountries_ASTER_SRTM.csv and AllCountries_ASTER_SRTM.sav that include the dataset in csv and SPSS format, respectively. In addition, there is a folder titled “countries” which contains country-specific.csv and .sav files. We also include an Excel spreadsheet that automates and aids the creation of new variables in SPSS (Supplementary Table 2).

### Extracting diverse fine-grained biogeophysical indicators through geospatial modelling

*Ecolo-zip* contains data of geolocation, as well as the means of elevation, normalized difference vegetation index, and land surface temperature and their respective standard deviations across observation time (2000–2008), and a land-water mask (i.e., the percentage of water coverage across a given surface area). The following section will explain the aggregated variables featured in *ecolo-zip* in more detail. The order in which the *ecolo-zip* variables are presented reflects the data layers of the ASTER data source, on which most variables are based upon. Since the ASTER data set is widely used and well-documented, this order facilitates the understanding and comparison of the *ecolo-zip* variables for the reader who is familiar with the ASTER data set or wants to learn more about it. Supplementary Table 1 offers an overview and detailed descriptions of all variables.

#### Elevation, mountainousness and sea level

**Fig. 3:**
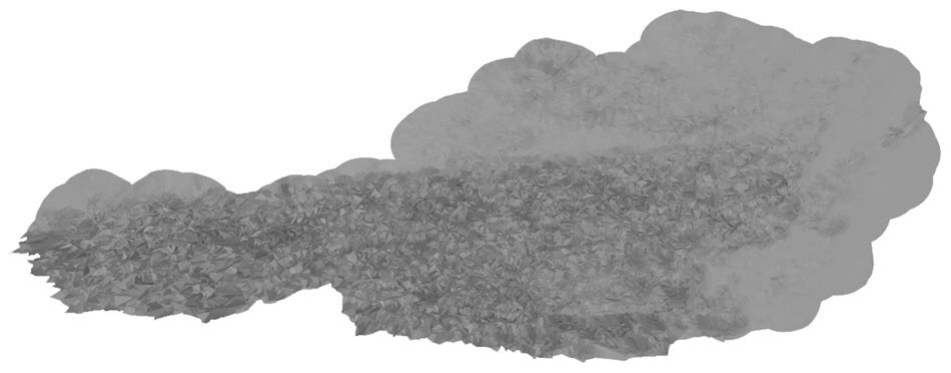
Example of overlaying geospatial models for SRTM elevation data in Austria. The country’s topology comprises the mountainous Alpine arc in the South and West and the Alpine foothills in the North and East. This distinct topology is well visible when overlaying all geospatial sampling models of all postal codes of the country.

A growing number of studies in a range of scientific disciplines point to an intricate relationship between geophysical topography and (a) human behavior^9^, (b) economic development^11^, and even (c) political systems^14^. In the *ecolo-zip* data, physical topography is operationalized through the two components of mountainousness (a) area elevation (altitude) and (b) hilliness (slope, shape) – in accordance with previous work^19^ and the Nordic Centre for Spatial Development^53^. This distinction is reflected in our measures of (a) the mean elevation capturing the average altitude and (b) the standard deviation of elevation capturing variability and shape – or in other words hilliness – of terrain within a postal code^54^. In addition, we provide the amount of data points at sea level (i.e., 0 meters) as a separate variable for both source datasets. In Fig. 2 we show two examples for how a mountainous region in the Austrian Alps and a flat region in the Italian Padan Plain are reflected in our geospatial sampling model. As an additional illustration, an overlay of the geospatial sampling models for all postal codes in Austria is shown in Fig. 3.

The variables included in the dataset are *SRTM_zip_elevation* and *ASTER_zip_elevation* with their respective mean, minimum, maximum, and standard deviation in *meters*, as well as the percentage of missing values (*missing_perc*, minimum = 0, maximum = 100), and the percentage of points at sea level (*cin*, minimum = 0, maximum = 100). For the sea level, values larger than one can be interpreted as a postal code being relatively closer (versus distant) to the sea.

#### Normalized difference vegetation index

The normalized difference vegetation index (NDVI) is well established in the scientific literature for providing a good representation of vegetation growth and phenology^55^ and constitutes a suitable measure of vegetation characteristics over time. It finds applications in ecological research, for instance in the contexts of gauging carbon cycles, global land cover maps^56^, land surface temperature^57^, and the leaf index^58^. The NDVI is closely related to the photosynthetic active radiation absorbed by biomass. It allows to differentiate between areas with dense vegetation such as tropical forest, areas with moderate vegetation such as grasslands and shrubs, and arid areas like sand, rocks, or concrete.

**Fig. 4:**
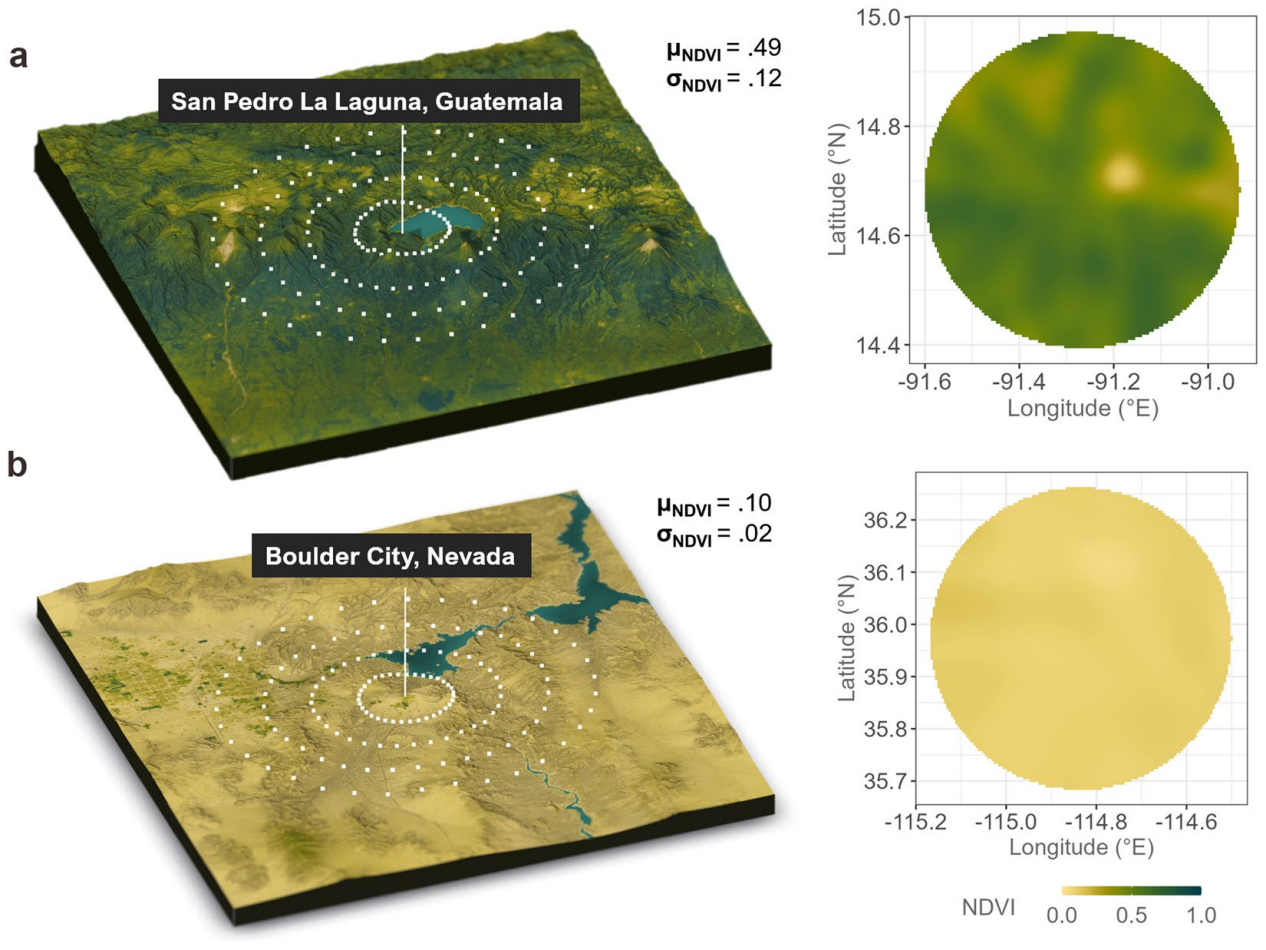
Sampling of normalized difference vegetation index (NDVI). The sampling of NDVI values in (**a**) the densely vegetated jungle of San Pedro de Laguna, Guatemala, and (**b**) the arid regions around Boulder City, NV, USA. White points reflect the estimated latitude and longitude for the geospatial sampling model.

The NDVI is calculated from the ratio of the *difference* versus the *sum* of surface reflectance in the near-infrared and red portions of the electromagnetic spectrum, is unitless, and ranges from +1.0 to −1.0. Dense vegetation such as tropical forest is reflected in high NDVI values (>0.6) whereas grasslands and shrubs commonly show values between 0.2 to 0.5. Areas with sand, rocks, or concrete show very low NDVI values 0.1 or less. Lastly, areas of water typically show negative values approaching −1. If red reflectance is increased due to atmospheric effects (e.g., clouds), the NDVI can be artificially reduced. When using the *ecolo-zip* dataset, it is critical to note that it employs top-of-atmosphere (TOA) reflectance and *not* atmospherically corrected surface reflectance. Note that atmospheric correction is not always needed and depends on the spatial resolution, sensor properties, and atmospheric conditions, e.g., water vapor, ozone, carbon dioxide. They are usually complex – especially on a global scale – and depend heavily on the quality of ancillary data. Nevertheless, ASTER-derived NDVI values show generally good agreement with different other NDVI-assessing instruments such as the Moderate Resolution Imaging Spectroradiometer^59^. Examples of densely vegetated and arid regions are shown in Fig. 4.

For each postal code, we aggregated the mean and the standard deviation for NDVI of the sampling points. The variables in the final dataset encompass *ASTER_ndvi_mean* (postal code-aggregated mean over the observation period between 2000 and 2008, values scaled from 0 to 100) and the respective minimum, maximum, standard deviation, and percentage of missings (*missing_perc*, minimum = 0, maximum = 100) across sampling points. In addition, we include postal code-aggregated standard deviations of the NDVI over time (i.e., *ASTER_ndvi_sd*), together with its mean, minimum, maximum, and standard deviation.

Please note that *ASTER_ndvi_sd* is used primarily as a technical variable for validation purposes. Relatedly, *ASTER_ndvi_sd_min* corresponds to the minimum value in the geospatial model sampling from the NDVI standard deviations across observations (i.e., across time from 2000–2008), *ASTER_ndvi_sd_max* corresponds to the maximum value of NDVI standard deviations among the sampling points. Although these variables might have only little practical relevance for researchers, we believe it is important to provide these values for completeness.

#### Land surface temperature

Land Surface Temperature (LST) is a pivotal environmental index of the earth climate system and land-atmosphere interactions measured by the thermal reflectance (i.e., the so-called *radiative skin temperature*). Investigating variations of LST has significance in broad research domains such as climate and vegetation research^11^ or social sciences^30,31^ where applications of large-scale satellite remote sensing have been continuously growing. Surface temperature is estimated from the mean emissivity between the years 2000 and 2008 from the cloud-free pixels using the Temperature Emissivity Separation algorithm. Thus, the mean LST reflects the average surface temperature during data acquisition in this time window and across years and seasons.

For each observation in the source data, the temperature has been averaged for each grid cell across time. For each postal code, we aggregated the mean and the standard deviation for temperature of the sampling points. Note that because temperature is subject to variability throughout the day, temperature values of postal codes with only a low number of average observations (<4) should be used with caution^60^. With this in mind, for ease of use, *ecolo-zip* features a specific binary variable that indicates whether the average observation number of a given postal code is smaller than four (*ASTER_observation_number_lt_4*).

The final dataset encompasses *ASTER_temp_mean* (postal code-aggregated mean over time, values range from 24,686 to 35,076 *Centikelvin [cK]*) and the respective minimum, maximum, standard deviation, and percentage of missings (*missing_perc*, minimum 0, maximum 100) across sampling points. The temperature variable can be transformed into degree Celsius [°*C*] by using the formula $[{}^{\circ }C]=\frac{[{}^{\circ }cK]}{100}-273.15$. In addition, we include postal-code aggregated standard deviation over time *ASTER_temp_sd*, with its mean, minimum, maximum, standard deviation, and missings (*missing_perc*). Analogously to the *ASTER_NDVI_sd* variable, *ASTER_temp_sd* is primarily a technical variable that we retained for validation purposes. *ASTER_temp_sd_min* corresponds to the minimum value in the geospatial model sampling from the Land Surface Temperature standard deviations across the observation period.

#### Land-water mask

The land-water mask – a measure of the percentage of water coverage across a given surface area – gives an estimation on whether a pixel in the source dataset corresponds to water from the original dataset (water = 1 and land = 0). It is a byproduct of the Temperature Emissivity Separation algorithm and calculated using visible bands only and, thus, based on reflectance from the top of the atmosphere.

We provide the average across the 20-mile area around a given postal code’s centroid (i.e., the sum of sampling points corresponding to land). The aggregated values correspond to the amount of freshwater or sea in the postal code. Higher values correspond to postal codes with plenty of water areas, lower values reflect areas with only little freshwater or sea. The dataset includes the variable *ASTER_lwm_water_perc* that includes the amount of sampling points corresponding to water surface (values range from 0 = *no points* to 100 = *all points* correspond to water).

#### Observations

The variable *ASTER_observations* provides the number of average cloud-free images acquired between 2000 and 2008 that were used to calculate the sampling point in the source dataset. Values range from 0 to 115 observations. We provide the postal code-aggregated values mean, standard deviation, minimum, and maximum. This acts as a quality control variable for users of this dataset.

#### Accuracy

*Geonames.com* includes a proprietary set of values to code the accuracy of the data source for the retrieved coordinates (anchored at 1 = *low*; 6 = *high*) that we included for each postal code. The *accuracy* variable corresponds to the precision of latitude and longitude estimated for each postal code. The values provided by *geonames.com* encapsulate the degree of precision for the supplied geo-coordinates and ranges from 1 ( = estimated from postal codes in numerical vicinity; least accurate) to 6 ( = the centroid of the geographical shape of the postal area; most accurate). Accuracy values of 2 and 3 indicate places with different names within the same postal codes (e.g., resulting from generalized area codes). Accuracy values of 4 and 5 indicate place names coming from the *geonames.com* database (rounded and exact coordinates).

The final aggregated data file is provided in a comma-separated value (csv) and a SPSS (sav) file. It includes postal codes of 94 countries and regions and six corresponding environmental variables based on ASTER Global Digital Elevation Map (GDEM) and Global Emissivity Dataset, and the SRTM DEM. Based on the geospatial model the environmental variables comprise postal-code aggregated values for diverse indicators of (1) physical topography (i.e., elevation, mountainousness, distance to sea; *ASTER_zip_elevation, SRTM_zip_elevation*), (2–3) climate (i.e., mean and standard deviation of land surface temperature; *ASTER_temp*), (4–5) vegetation (i.e., mean and standard deviation of the NDVI; *ASTER_ndvi*) and (6) blue spaces (i.e., the land-water mask, approximating surface water coverage of an area; *ASTER_lwm*). In addition, we provide the gazetteer data of *geonames.com* including administrative names (country, region, municipality), estimated postal code centroid latitude and longitude (coordinates in WGS84) and their respective postal code accuracy, as well as the difference between SRTM and ASTER GDEM. All variables included in the dataset are summarized in Supplementary Table 1.

## Technical Validation

**Fig. 5:**
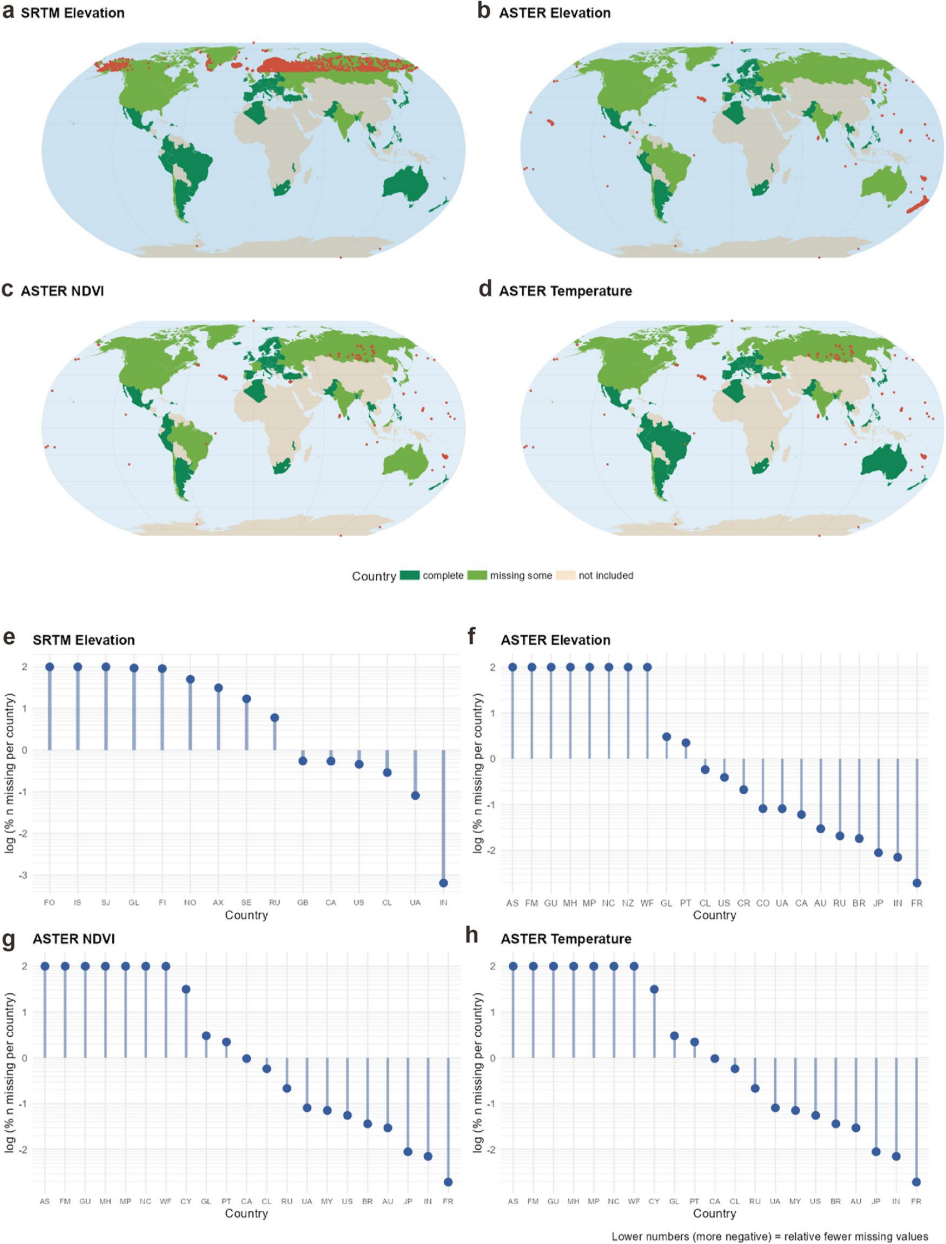
Data coverage. (**a**–**d**) Visualizations of missing data points (in red) across the globe for the main aggregated variables. Missing data points were mostly found in maritime regions with islands. In the SRTM dataset (**e**) countries north of 60°N were missing completely or significantly (e.g., Iceland or Finland). (**f**–**h**) The missing datapoints from the SRTM source were included in the ASTER source, however, ASTER has more missings in countries with large coastal areas or small islands (e.g., parts of Portugal or Canada).

In principle, the ASTER data include data points across latitudes between 83°N and 83°S where enough valid measurements of the ASTER instrument were available. In particular, the dataset includes most countries in Europe, most of Central and South America, North America, Oceania, and various countries from Africa and Asia. We summarized the included countries along with their respective data availability in Supplementary Table 3. Note that for some countries a small number of datapoints are not available. This applies primarily to maritime areas and small islands. Data availability for ASTER Global Emissivity Dataset and GDEM, as well as the SRTM GDEM is summarized Fig. 5.

The ASTER Global Emissivity Dataset source data has been validated in several independent studies^59,61^. More specifically, Miura and colleagues (2008) found convergent values for ASTER and the widely used Moderate Resolution Imaging Spectroradiometer NDVI measurements. The small deviations between the two measurements that do exist have been attributed to differences in the underlying imaging methods (e.g., ASTER uses a wider spectral band) or atmospheric correction profiles (e.g., aerosol correction is not applied in the ASTER dataset).

The LST and its retrieval via the Temperature Emissivity Separation algorithm have been validated in several studies^62–65^ and achieve a precision of approximately 1.0–1.5 Kelvin for most scenes with accurate measurements^66^ depending on the conditions of the land surface (e.g., vegetation or moisture). This reflects a relatively accurate measurement of land surface temperature and good comparability. The Jet Propulsion Laboratory reports different emissivity profiles of (pseudo-)invariant sand dune sites comparing ASTER data with *in-situ* measurements directly on their website (https://emissivity.jpl.nasa.gov/validation), showing that the difference is usually smaller than 2%.

### Comparison of ASTER and SRTM

In regions where the elevation models of ASTER and SRTM overlap – namely across latitudes between 60°N and 56°S – we employed the SRTM dataset for comparison and validation of the main ASTER dataset. The difference in meters between the SRTM and ASTER elevation models was included in the final dataset as a new variable (*Difference_elevation_mean_SRTM_ASTER*, Supplementary Table 1).

**Fig. 6:**
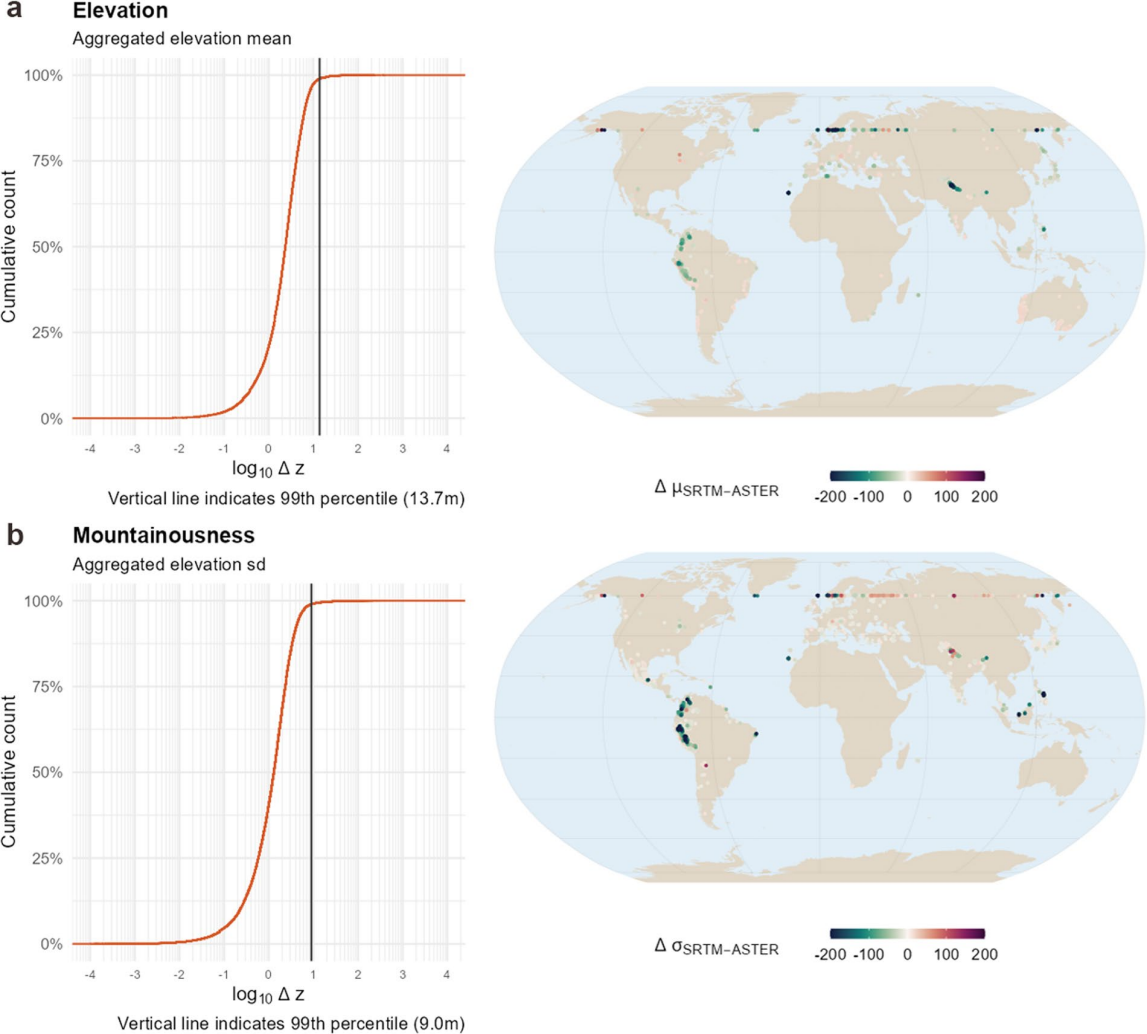
Data validation between SRTM and ASTER. (**a**) Aggregated elevation values from postal codes were highly consistent across datasets in most regions with a 99th percentile of deviation of 13.7 m. As a general pattern, SRTM values were slightly higher on average, which is in line with previous research that shows overestimation of SRTM and underestimation of ASTER elevation values. Largest deviations were found in the North Andes and the Himalaya, particularly mountainous areas where SRTM acquisition has been difficult due to the mountain slopes. (**b**) Unsurprisingly, mountainousness (SD of elevation) showed also high consistency across measurement methods and showed largest deviations in the same areas. The 99th percentile of the deviations comprised 9.0 m.

The largest differences were found in mountainous areas with high elevation, particularly in the Himalaya and the Northern Andes (Fig. 6). In comparison, we found that the SRTM showed lower aggregated elevation values than the ASTER. A reason for this could be that the measuring method of the SRTM satellite is creating larger measurement errors in areas with steep slopes (>10°) than the ASTER instrument^33^. Previously reported higher accuracy of SRTM in flat areas which are often associated with agricultural or (more) vegetated land and underestimation of elevation by ASTER was negligible at the aggregated level. In general, we found acceptable differences in elevation (99^th^ percentiles of Δ *z* = 13.6 m for elevation and Δ *z* = 9.0 m for mountainousness; Fig. 6). The Intraclass-Correlation Coefficient (ICC) was 0.999, while the Pearson correlation was 1.0 (*p* < 0.001). Although the results were largely comparable and the usage of aggregated ASTER elevation values can be recommended generally, in densely vegetated regions SRTM data might benefit from the radar interferometry which can penetrate tree canopy and should translate into more accurate measures in this context. Thus, in this specific case, it might be advisable to fall back to the elevation metrics calculated from the SRTM source. We acknowledge that both ASTER and SRTM have their own advantages and limitations depending on the context and purpose of the analysis, and we provide both sources separately in our dataset to offer more flexibility and choice to users who can select the most suitable source for their research question or combine them in a way that maximizes their strengths and minimizes their weaknesses.

## Usage Notes

To the best of our knowledge, the present dataset – *ecolo-zip* – is the first to offer detailed topological and environmental satellite data – and thus a rich, granular description of humans’ natural ecology – for over 1.5 million postal codes across 94 countries and regions using high-resolution datasets that can potentially enrich the investigations of many research fields. The aggregations are based on two large-scale satellite image data resources (ASTER, SRTM), each with a spatial resolution of ≈100 m, offering an accurate source layer for the geospatial sampling model and mitigating residual errors. The aggregated variables are available for use as predictors in environmental or topographical studies to model influences of mountainousness, vegetation, or surface temperature for research questions involving geolocation data to assess the impact of environmental factors in fine-grained spatial areas and administrative units. The fact that ASTER data provides almost global coverage allows researchers to conduct timely comprehensive analyses and adopt a *zoomed-out*^67^ perspective on the many ways in which environmental variables are related to human life and behavior across countries, continents, and cultures. By using our dataset as a proxy for different habitats and climates around the world, researchers can test different theoretical perspectives on how humans adapt to their ecological context. Furthermore, our dataset allows researchers to integrate zoomed-out approaches with zoomed-in approaches by combining these data with other forms of data – for instance, social, political, economic, or health related indicators which are readily available in many countries at various levels of spatial aggregations^68^. We firmly believe that – in conjunction – this has strong potential to promote exciting avenues for future work across diverse research fields.

Nevertheless, when using the present dataset, researchers should be mindful of challenges posed by the underlying source datasets. First, a limited number of accuracy restrictions for the geo-coordinates of postal codes in this dataset apply in certain countries. For Canada, Chile, Ireland, and Malta the dataset includes only the first letters for copyright restrictions of the data providers. For Argentina and Brazil, only the major postal codes for each municipality are included. That is, only postal codes ending in −000 for Brazil, or the first five letters/numbers for Argentina.

Second, SRTM elevation data are only available for latitudes between 60°N and 56°S, meaning that some regions in the northern hemisphere were not captured in that dataset. However, various validation studies demonstrate that the ASTER Global Digital Elevation Map still reaches acceptable error levels beyond these extreme latitudes^61,69^ corroborating that the provided ASTER data meet desired baseline accuracy level. Moreover, our results show that among latitudes close to 60°N there seem to be particularly high deviations between the SRTM and ASTER elevation data that users of this dataset should be aware of. We suggest using ASTER elevation data in these northern regions, due to the broader coverage of the underlying source data. Nonetheless, because of the different methods in generating the elevation models, minor deviations in quality are to be expected in these – less densely populated – latitudes. A possible extension in further research is to employ population weighting, in which each postal code receives a weight proportional to its population size. This might be especially desirable when studying areas with uneven population density, such as countries wherein inhabitants cluster in a handful of highly urbanized areas while large swaths of the rest of the country is sparsely populated.

Lastly, especially in some maritime and mountainous regions, some postal codes did not include enough valid data points to aggregate the information. In most instances, this is likely due to clouds in combination with low observation numbers at the time of source data collection. Therefore, the data of these postal codes could not be included in our final dataset (~0.4%). Notwithstanding these limitations, we believe that the current resource – featuring a diverse array of core biogeophysical indicators for over 1.5 million postal codes around the world – has the potential to enrich many different strands of research at the intersections of the physical and social sciences. By sharing it freely, we hope to contribute to a better understanding of the multi-faceted interactions between humans and their environments – and empower others to do the same.

### Supplementary information

Supplementary Table 1

Supplementary Table 2

Supplementary Table 3

## Data Availability

The code written and used for the data collection is available in the repositories specified in the references. Furthermore, we provide an Excel sheet to aid users to automatically create SPSS syntax to add new variables to the dataset. Programs used, including versions, are outlined in the repository descriptions.
